# Harnessing CRISPR potential for intervertebral disc regeneration strategies

**DOI:** 10.3389/fbioe.2025.1562412

**Published:** 2025-05-08

**Authors:** Catarina Milheiro, Maria L. Moura, Mario Amendola, Mário A. Barbosa, Joana Caldeira

**Affiliations:** ^1^ i3S – Instituto de Investigação e Inovação em Saúde, University of Porto, Porto, Portugal; ^2^ ICBAS - Instituto de Ciências Biomédicas Abel Salazar, University of Porto, Porto, Portugal; ^3^ Généthon, Évry, France; ^4^ Integrare Research Unit UMR_S951, Université Paris-Saclay, Université Evry, Inserm, Généthon, Évry, France

**Keywords:** CRISPR/Cas9, intervertebral disc, low back pain, degenerative disc disease, genome editing tools

## Abstract

Genome editing technologies, particularly CRISPR (Clustered Regularly Interspaced Short Palindromic Repeats), have broadened the possibilities of genetic research and molecular biology by enabling precise modifications of the genome, offering novel therapeutic potential for various disorders. Herein, we present an overview of traditional genome editing techniques and delve deeper into the CRISPR toolbox, with particular attention given to epigenetic and transcriptional regulation. In the context of the intervertebral disc (IVD), CRISPR offers an unprecedented approach to address the mechanisms underlying tissue degeneration, advancing the development of revolutionary therapies for Low Back Pain (LBP). As so, we showcase how to leverage CRISPR systems for IVD. This cutting-edge technology has been successfully used to improve our understanding of IVD biology through functional studies and disease modeling. Most relevant research prioritizes new targets associated with the extracellular matrix (ECM), pain sensing or inflammatory pathways. Promising CRISPR applications encompass IVD regeneration by recapitulation of a regenerative environment or by targeting important degenerative catalysts. In the future, priority should be given to fetal gene reactivation, multiple healthy gene expression enhancement and disease-associated polymorphisms’ correction. Despite several challenges such as effective delivery, off-target effects, as well as ethical and safety concerns, exciting clinical trials are anticipated in the years to come, providing more effective and long-lasting solutions for IVD degeneration.

## 1 Introduction

### 1.1 The intervertebral disc

**FIGURE 1:**
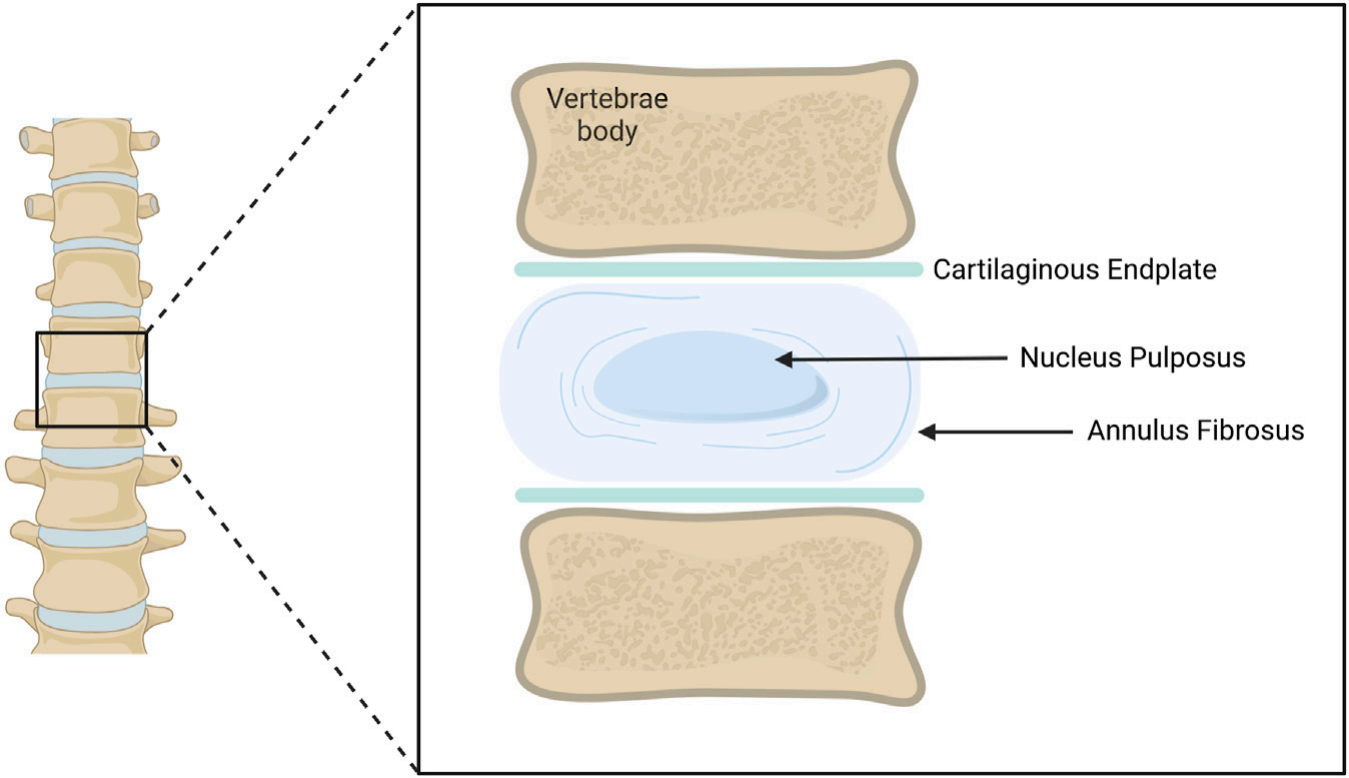
Schematic representation of an adult Intervertebral Disc (IVD). The IVD is located between vertebral bodies. The Annulus Fibrosus (AF) surrounds the Nucleus Pulposus (NP). Both structures are in contact with upper and lower cartilaginous endplates, separating them from the vertebrae. Illustration created using BioRender.

The intervertebral disc (IVD) is an avascular and aneural cartilaginous-like structure that exists between vertebrae enabling load distribution and spine mobility. It was first described by Wright *et al* in 1973 (Wright et al., 1973). The disc is organized into three main components: the inner nucleus pulposus (NP), its surrounding annulus fibrosus (AF), and the cartilaginous endplates (CEPs) which separate discs from vertebrae (Humzah and Soames, 1988; Walter et al., 2015). Each of these structures comprises distinct cell populations and matrix elements, tailored to their function (Figure 1).

The NP is mainly composed of water, proteoglycans (PGs) and randomly oriented fibers of collagen type II (Whatley and Wen, 2012). Despite hypoxic conditions and increased osmotic pressure, some cell populations are still able to survive, as small chondrocyte-like cells and large vacuolated notochordal-like subsets. Chondrocyte-like cells are crucial for extracellular matrix (ECM) production and deposition, while notochordal-like cells are associated with the NP embryonic origin (Pattappa et al., 2012). Altogether this structure and its components are responsible for resisting compressive forces and supporting spinal loads (Nachemson et al., 1979).

Deformation of the NP is restrained by the AF, that is composed of water, collagens and PGs. Collagen type I and type II fibers, present in higher amounts in the outer and inner regions of the AF, respectively, are organized in concentrically oriented lamellae (Shapiro and Risbud, 2014). Due to its fibers’ organization, each concentric AF layer is crucial for tissue mechanical behavior and the complex distribution of stress including resistance to tensile stress (Nerurkar et al., 2010). Fibroblast-like cells, displaying an elongated morphology are present in the outer AF, while chondrocytes can be found in the inner region (Whatley and Wen, 2012).

Lastly, the CEPs are composed of a thin layer of hyaline cartilage that is connected to the vertebral bodies (Eyre, 1979). Characterized by a matrix rich in aggrecans and collagen type II, these structures are responsible for the disc’s biomechanical support, preventing bulging of the NP against the vertebral bodies (Crump et al., 2023). Additionally, as the endplates are the closest structure to vascular capillary, they are critical for IVD nutrition (Rodriguez et al., 2011).

Due to its anatomy, the lack of vascularization and innervation in the IVD creates an immune-privileged environment. Additionally, the organized compartmentalization of each IVD structure, along with their morphology and composition, which result from the mechanical forces exerted during development, are crucial for maintaining homeostasis and tissue function (Walter et al., 2015). Disruption of ECM turnover and cellular function affects tissue microenvironment, leading to largely irreversible structural changes given the disc’s limited ability to regenerate.

### 1.2 Intervertebral disc degeneration and current therapeutic solutions

Throughout the degenerative process, changes in ECM structure, altered innervation and inflammation are thought to promote the onset of Low Back Pain (LBP). LBP is the leading cause of years lost to disability, affecting around 80% of the world population (Buchbinder et al., 2018). Due to its high prevalence, it has a huge socioeconomic impact due to costs associated with treatment and work absenteeism.

**FIGURE 2:**
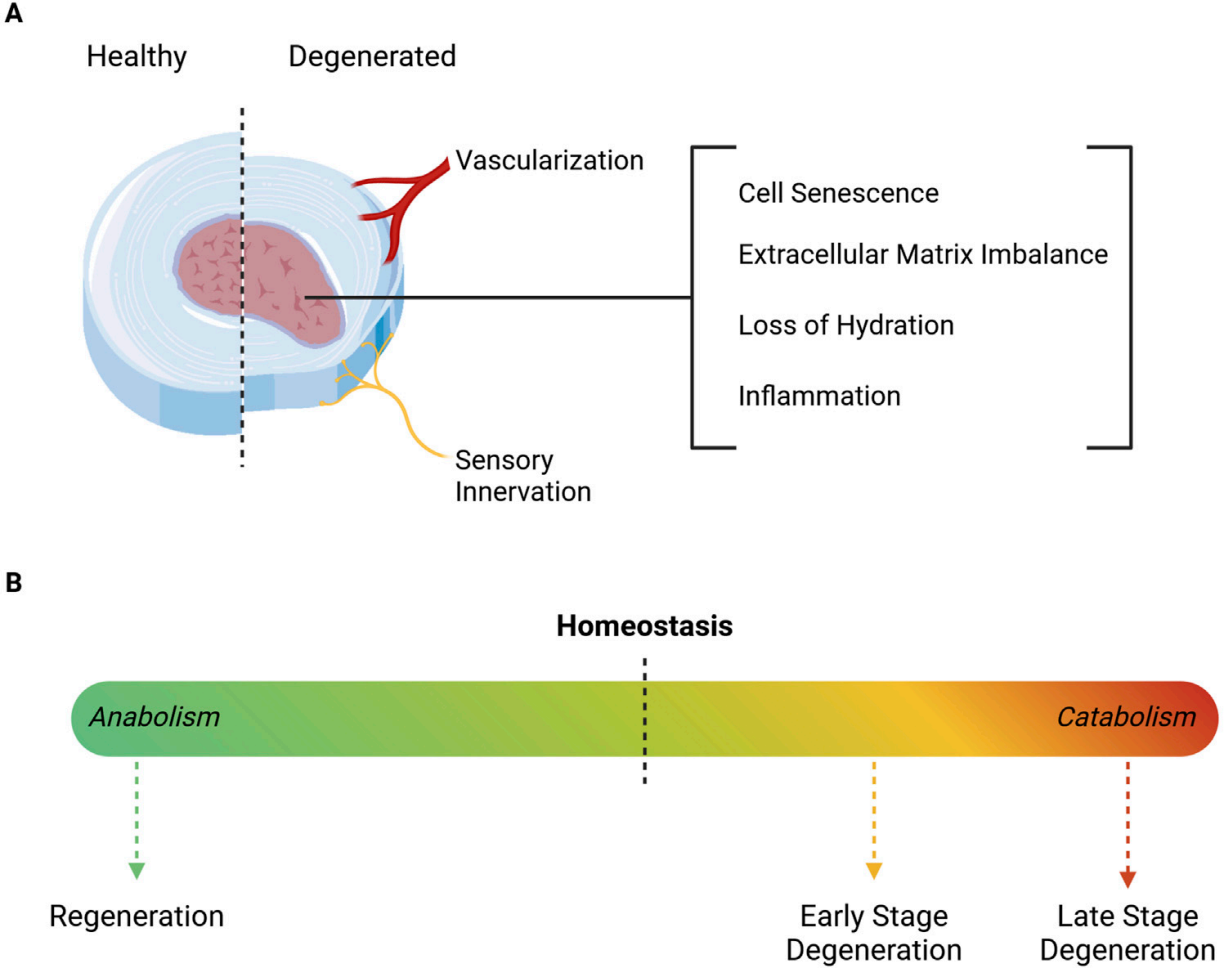
Schematic representation of degeneration-associated Intervertebral Disc alterations. **(A)** The degenerated IVD is characterized by inflammation, dehydration, cell senescence and ECM depletion, which altogether affect tissue structure and function. The degenerative cascade is accompanied by a reduction of disc height, along with the ingrowth of blood vessels and nerve fibers. **(B)** ECM turnover is crucial for maintaining homeostasis and ensuring proper mechanical function. However, as degeneration progresses, the microenvironment transitions to a catabolic state, marked by severe cell death and increased depletion of ECM components. In turn, regeneration requires a shift towards a more anabolic setting, in order to promote cell proliferation and ECM production. Illustration created using BioRender.

IVD degeneration is the most common cause of chronic LBP and can occur naturally with ageing, or as a pathological process, driven by an interplay between environmental and genetic factors (Kepler et al., 2013). Ageing-related alterations to disc structure progress at different rates among individuals. However, they consistently impair IVD integrity, due to a shift towards a more catabolic environment. This structural decline further contributes to IVD dehydration and height reduction (Zhao et al., 2007), as summarized in Figure 2. Once alterations on cell senescence and apoptosis, ECM production and degradation, neural and vascular ingrowth, and pro-inflammatory cues begin (Kepler et al., 2013; Novais et al., 2024), the discs are unable to restore homeostasis, which further contributes to long-term tissue deterioration.

Despite its multifactorial etiology, the pathological process known as Degenerative Disc Disease (DDD), is more strongly influenced by genetic factors than by environmental stimuli (Battié et al., 2009). Polymorphisms in genes encoding aggrecan, vitamin D receptor (Eser et al., 2010), collagen type IX (Jim et al., 2005), collagen type I (Pluijm, 2004) and matrix-metalloproteinases (Takahashi et al., 2001) have been associated with disc degeneration. Even though numerous triggers are involved in the degenerative cascade, the outcome tends to favor matrix degradation.

Throughout the degenerative process, matrix metalloproteinases (MMPs) increase the degradation of collagens and proteoglycans present in the ECM, affecting its natural turnover. This degradation of matrix components alters NP water content and structural integrity, decreasing the osmotic pressure and consequently, its biomechanical properties. As the NP becomes unable to support the spine compressive forces, the AF starts to weaken. Collagen type II is replaced by collage type I fibers, disrupting AF layers and causing structural damage over time (Whatley and Wen, 2012; Dittmar et al., 2016).

An additional degeneration-associated feature that greatly contributes to DDD is the increase in pro-inflammatory cytokines such as interleukin (IL)-1β, IL-6, IL-8 and Tumor necrosis factor (TNF)-α (Molinos et al., 2015). Upregulation of IL-1β and TNF-α production by AF and NP cells, further promotes ECM degradation and MMP production, contributing to a degenerative cascade and triggering discogenic pain (Hoyland et al., 2008). The expression of these pro-inflammatory molecules influences cell senescence and autophagy, both of which are characteristic of degenerated IVDs (Risbud and Shapiro, 2014; Silwal et al., 2023). Despite viable, cells undergo morphological and phenotypic changes, forming aggregates, increasing in size and developing vacuoles (Dowdell et al., 2017). This pro-inflammatory cell profile has been termed senescence-associated secretory phenotype (SASP) and can be promoted by various types of stimuli such as mechanical overload, oxidative, metabolic and inflammatory stress or impaired autophagy (Kim et al., 2009; Dimozi et al., 2015; Vamvakas et al., 2017; Kang et al., 2022). The increase of senescent cells with age and degeneration, also associated with telomeres degradation, hinders IVD homeostasis and negatively affects nearby cells (Song et al., 2023).

Several conditions might arise as a consequence of IVD degeneration including spinal stenosis, disc herniation and LBP (Moore et al., 1996; Cheung et al., 2009; Knutsson et al., 2015). Current treatments focus on conventional approaches like physical therapy, anti-inflammatories, analgesic drugs and muscle relaxants, which provide short-term symptom relief but fail to address the underlying degeneration process, often manifesting as persistent chronic pain (Romaniyanto et al., 2022; Samanta et al., 2023; Sono et al., 2024). Remarkably, painkillers, especially opioids, have been strongly discouraged due to minimal gains but high risks like overuse and addiction (Foster et al., 2018). In more advanced cases, invasive surgical interventions like spinal fusion or disc arthroplasty are employed. While these methods can stabilize the spine and alleviate nerve compression, they frequently lead to comorbidities, infections and complications such as adjacent segment degeneration, being unable to restore the normal structure or function of the IVD and ultimately leading to prostheses wear and reinterventions (Romaniyanto et al., 2022; Sono et al., 2024).

Emerging regenerative therapies, including tissue engineering, growth factor administration, gene therapy and cell-based treatments are also being explored, aiming to reverse degeneration and restore disc homeostasis (Huang et al., 2016), but not without drawbacks. In the last decades, several biomaterials arose, but only a handful made it to the clinics. Natural hydrogels do not meet biomechanical requirements, while synthetic ones have poor biocompatibility (Desai et al., 2024; Wachs et al., 2017). The use of decellularized IVD scaffolds, from animal origin, arose in the context of IVD regeneration, as a way to preserve native tissue structure and composition. Still, they are challenged by batch-to-batch variability, zoonosis, donor age and availability (Fiordalisi et al., 2020; Fiordalisi et al., 2021; Illien-Junger et al., 2016). Strategies based on growth factors are also being investigated, but multiple injections are required to ensure a lasting effect. Gene therapies have also been proposed but continue to raise several ethical and safety issues. In turn, cell-based therapies using mesenchymal stem cells (MSCs) have shown promise in reducing inflammation and promoting extracellular matrix synthesis, with several clinical trials currently ongoing (Binch et al., 2021). However, challenges such as poor homing and survival in the harsh IVD microenvironment, limited long-term efficacy and cell leakage still remain (Vadalà et al., 2012; Loibl et al., 2019). Hence, there is an urgent need to develop novel clinical solutions that recapitulate healthy tissue, particularly those that can improve the already existing cell-based therapies.

Clustered regularly interspaced short palindromic repeats (CRISPR) technology provides a transformative solution by enabling precise genetic modifications to target key drivers of regeneration, providing long-lasting therapeutic effects with a single intervention (Samanta et al., 2023). By addressing the molecular mechanisms of the disease, it holds the potential to shift LBP treatment paradigm from symptom management to sustained regeneration and functional restoration. Moreover, when comparing CRISPR-mediated transcriptional activation or repression, to traditional genome engineering, there are additional benefits as physiological gene expression/silencing, reversible effects, simple target design, genome-wide access, and predictable off-target effects and multiplexing (Sun et al., 2016). These reasons underscore the rationale for centering this review on the underexplored yet promising therapeutic potential of CRISPR-based strategies in the context of LBP and IVD regeneration.

## 2 An overview of CRISPR/Cas systems

CRISPR sequences were first discovered in prokaryotic genomes as part of their adaptative immune system. Initially, the function of such distinct sequences was unknown, but further studies to understand their mechanism of action and function shed light on their potential applications (Barrangou et al., 2007). Adapting this technology for mammalian cells and *in vivo* gene editing was a breakthrough that led researchers from a variety of fields to adopt this technique (Jansen et al., 2002; Doudna and Charpentier, 2014), thus leading to its broad recognition with a Nobel Prize in 2020.

The CRISPR system consists of an endonuclease that is able to specifically target (by sequence complementarity) and cleave deoxyribonucleic acid (DNA) by the action of a single strand guide ribonucleic acid (sgRNA) (Cong et al., 2013; Jinek et al., 2012). There is an amazing diversity of CRISPR systems, classified based on the CRISPR-associated (Cas) genes structure, as different Cas proteins have specific recognition sites and cleaving abilities. Two main classes have currently been described: Class 1, found in the majority of bacteria and archaea (around 90%), is characterized by multiprotein effector complexes, operated by different Cas nucleases; Class 2, where the effector complex consists of a single protein (Hillary and Ceasar, 2023; Makarova et al., 2015). Moreover, there are different system types within each class, depending on its components mechanism of action. Currently, Class 1 systems are organized into type I, III and IV, corresponding to the effectors Cas3, Csm6/Cmr complex or Cas10 and Csf4. Class 2 systems can be categorized into type II, V and VI with the effectors being Cas9, Cas12/14 and Cas13, respectively (Su et al., 2023). The different systems are described in Table 1. As the field evolves, some rare systems are being discovered but have yet to fit into the ongoing classification. Additionally, it is worth noting that in both classes, within each type, there are subtypes that fall out of the scope of this review.

**TABLE 1:** Classification of identified CRISPR/Cas systems.

Class	1			2		
Type	I	III	IV	II	V	VI
Effector	Cas3	Csm6/Cmr Complex and Cas10	Csf4	Cas9	Cas12 (Cpf1) and Cas14	Cas13 (C2c2)
Target nucleic acid	ssDNA	RNA and ssDNA	—	dsDNA, ssDNA, ssRNA	ssDNA, dsDNA	ssRNA

ss: single strand/ds: double strand.

CRISPR/Cas9 is the most widely used system. This type II system is composed of a Cas9 endonuclease (commonly derived from *Streptococcus pyogenes*, SpCas9) and sgRNA. The engineered RNA is designed to target a specific 20 base-pair (bp) sequence of genomic DNA. For the system to work, the target sequence must be flanked by an upstream sequence termed “protospacer adjacent motif” (PAM) (Anders et al., 2014). The necessary and recognizable PAM sequence depends on the Cas protein used. Cas9 for instance recognizes and acts before an “NGG” or “NAG” PAM sequences, favoring “NGG,” while Cas12 recognizes thymine-rich PAM sequences. Altogether, in the CRISPR/Cas9 system, the 20bp sgRNA directs the Cas9 binding to the target DNA (Anders et al., 2014; Sander and Joung, 2014). The endonuclease cleaves the region of interest and generates a double-strand break (DSB), which allows us to introduce the desired modification ranging from insertion of single nucleotides or entire genes to deletions, or even substitutions.

The enormous impact of CRISPR/Cas technology in the last years has been a direct consequence of its advantageous characteristics when compared to the more traditional genome editing tools with reduced associated costs. From its initial use for mutation correction in hematopoietic disorders like sickle cell disease (Dever et al., 2016), to inflammation and microenvironment modulation (Brunger et al., 2017), the constant developments have given scientists the opportunity to edit virtually any gene, in a wide array of cell types and even more complex *in vivo* models. This technology is strengthened by the ability to simultaneously target multiple loci, referred to as multiplex (Kabadi et al., 2014; Konermann et al., 2014); the development of drug inducible systems, like doxycycline-induced Tet system (González et al., 2014; Aubrey et al., 2015), or drug control of Cre recombinase activity (Roper et al., 2017; Oldrini et al., 2018); and the more recent development of base editing (Brusson et al., 2023) and prime editing (Anzalone et al., 2019) approaches.

However, despite current advances, the use of CRISPR still faces limitations, mainly regarding its delivery into target cells, off-target effects, immunogenicity and PAM sequence restrictions. In addition, it has been a hot topic in ethical discussions, mainly regarding germline cell and embryo editing (Ayanoğlu et al., 2020). Over time, with continuous fine-tuning and wider applications, CRISPR has gained an undeniable significance for biological research, being used in basic, preclinical and clinical studies. A recent review describes the current registered clinical trials using CRISPR-based strategies, most of which are focused on cancer and hematopoietic disorders, in phase one or phase two (Zhang et al., 2023). Of note, a critical milestone in CRISPR translation was achieved in the end of 2023, with both the U.S. Food and Drug Administration (FDA) and the European Medicines Agency (EMA) approving Casgevy, the first CRISPR-based therapy for the treatment of sickle cell disease and β*-*thalassemia (European Medicine Agency, 2023; U.S. Food and Drug Administration, 2024). Briefly, this therapy consists of injecting CD34^+^ hematopoietic stem cells that have been engineered *in vitro* using the CRISPR/Cas9 system to disrupt the erythroid specific enhancer of the *BCL11A* (B-cell lymphoma/leukemia 11A) transcriptional repressor gene, thereby reducing its expression/activity and allowing for the re-expression of the silenced fetal hemoglobin. Nonetheless, other CRISPR-based strategies have been developed to address these severe hereditary blood disorders. Pavani and colleagues, for instance, combined two approaches to correct the α/β-globin imbalance in β-thalassemia patients (Pavani et al., 2021) by using CRISPR/Cas9 to delete *HBA2* (Hemoglobin Subunit Alpha 2) gene and simultaneously insert a β-globin transgene. This dual targeting showcases CRISPR/Cas9 potential when tackling complex genetic disorders.

In the last decade, the versatility of CRISPR technology has enabled its application to various research fields. Repurposing for tissue engineering and regenerative approaches has been thoroughly reviewed (Hsu et al., 2019; Razavi et al., 2024), with various recent reviews focused on promoting cartilage regeneration (Graham et al., 2023; Li et al., 2023; Jia et al., 2024). However, a tissue that has been overlooked is the IVD. Indeed, the use of CRISPR for IVD studies is still in its early stages revealing the untapped potential of the field. In this review we discuss the most recent advances of CRISPR use beyond its molecular scissors function exploring how this toolbox can be applied to better understand IVD pathobiology and to unveil novel regenerative strategies targeting this tissue.

### 2.1 The CRISPR toolbox for transcriptional and epigenetic modulation

The engineering of Cas proteins for gene regulation without base-pair modifications expanded the panoply of CRISPR applications, triggering research on new fusion proteins able to activate and repress gene expression or perform histone modifications.

The development of an inactive nuclease, the dead Cas9 (dCas9) mutant, has been crucial for redirecting CRISPR as a tool to regulate transcriptional and epigenetic processes (Qi et al., 2013). A sequence specific non-mutagenic Cas9, was obtained by two mutations in the endonuclease domains HNH and RuVC1, more specifically in the H840A and D10A residues, respectively (Jinek et al., 2012). Despite the different strategies available, most of the CRISPR/dCas9 systems are organized into three components: a) dCas9; b) sgRNA; c) effector domain (transcriptional or epigenetic modulators). Briefly, dCas9 is guided by a sgRNA, forming a sgRNA/dCas9 complex, that specifically binds to the DNA without cleaving it (Qi et al., 2013).

The versatility of the system lies in the possibility to use the dCas9 alone or pair it with different effector domains, targeting the system to the promoter/enhancers regions of the genes of interest and either activating (CRISPR activating) or impairing (CRISPR interference) gene expression. Moreover, the dCas9 can also be fused with epigenetic modulators for targeted regulation of genome loci. These systems have domains that can alter chromatin marks such as DNA methylation or histone modifications by recruiting epigenetic effectors and regulating gene expression (Adli, 2018). Usually, methylation of the promoter is responsible for gene silencing whereas histone modifications might, depending on target, lead to gene expression activation or repression. For instance, histone H3 lysine 4 residue acetylation or tri-methylation leads to gene activation, while histone H3 lysine 4 di-methylation silences gene expression (Gibney and Nolan, 2010).

Proper sgRNA design is crucial for system efficiency. As previously mentioned, the presence of an NGG PAM sequence in the desired target region is a requirement. An additional consideration is that the sgRNA must target regulatory regions of the gene upstream of the transcriptional starting site (TSS) which might not be annotated in the genome. The presence of alternative promoters, the epigenetic landscape and the presence of DNA binding proteins in the target site can also interfere with sgRNA design and/or binding. The risk of off-targets in the genome led to the development of strategies relying on the use of different Cas homologous, like Cas12a (Cpf1), which can recognize larger PAM sequences, thus increasing specificity (Huang et al., 2023). Mutated forms of Cas12 have been developed for transcriptional regulation studies but have been mainly applied to prokaryotic or plant models (Tak et al., 2017). In the scope of this review, we will focus on dCas9-based CRISPR activating and CRISPR interference systems, as well as their mechanism of action.

#### 2.1.1 CRISPR activation systems

Gene expression activation is possible with an approach designated as CRISPR activating (CRISPRa). The conventional method of introducing exogenous DNA for gene overexpression results in the incorporation of multiple copies of the same gene. In contrast, employing dCas9 activators to induce gene expression leads to more biological expression levels. Nonetheless, the activation levels will vary depending on the cells’ basal expression levels, epigenetic landscapes and sgRNA target sites.

**FIGURE 3:**
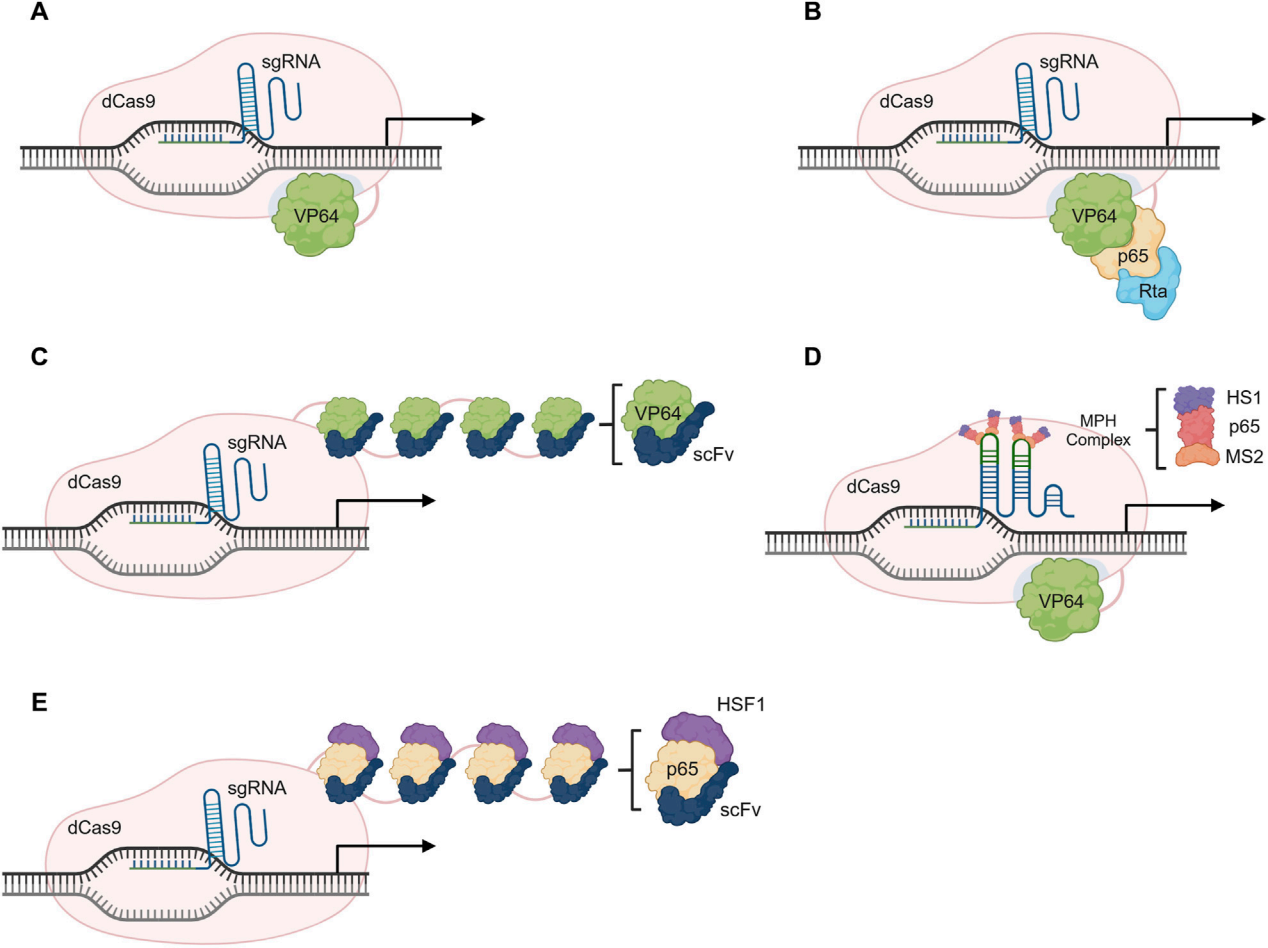
Overview of CRISPR activating (CRISPRa) systems. **(A)** Gene activation using the dCas9-VP64 system. **(B)** The dCas9-VPR system consists of a fusion protein with the transcription activators VP64, p65 and Rta (VPR). **(C)** The dCas9-SunTag relies on the action of multiple VP64 molecules that are in a tandem of GCN4 peptide repeats tagged with a single-chain variable fragment (scFv) of the anti-GCN4 antibody. **(D)** Gene activation using dCas9-SAM relies on VP64, p65 and HSF1 activators, and requires the recruitment of the MCP-p65-HSF1 (MPH) complex through MS2 loops present in the sgRNA. **(E)** The dCas9-SunTag-p65-HSF1 uses the structure of SunTag system but relies on the p65 and HSF1 activators instead of the VP64. Illustration created using BioRender.

The initial system developed was the dCas9-VP64, which relied on the fusion of the dCas9 with VP64, a transcriptional activator. In the original paper, a pool of 22 sgRNAs targeting either the *VEGFA* (Vascular Endothelial Growth Factor A) or the *NTF3* (Neurotrophin 3) regulatory regions was assessed. Despite observing great variability, 21 out of the 22 sgRNAs were able to induce gene upregulation (Maeder et al., 2013). To boost its potential, several sgRNAs targeting the same gene can be used simultaneously, in a multiplex approach. Additional systems have been developed to promote stronger activation, while using only one sgRNA and are described in the following subsections, namely, the dCas9-VPR (Chavez et al., 2015), the dCas9-SunTag (Tanenbaum et al., 2014) and the dCas9-SAM (Konermann et al., 2014) systems (Figure 3).

##### 2.1.1.1 dCas9-VPR system

The dCas9-VPR (Figure 3B) is composed of a dCas9 paired with a tandem fusion of VP64, the activation domains of the p65 subunit of NFκB (Nuclear factor kappa B), and Epstein-Barr virus R transactivator, Rta. The complex is guided to a region upstream of the TSS and the VP64-p65-Rta effector domain is responsible for the recruitment of transcription factors that stimulate gene expression (Chavez et al., 2015).

##### 2.1.1.2 dCas9-SunTag system

A strategy to amplify a regulatory signal is to recruit several copies of the effector protein to the desired target. The dCas9-SunTag (Figure 3C) relies on the recruitment of multiple VP64 domains to enhance transcriptional activation. For that, the system consists of a tandem of GCN4 peptide repeats tagged with a single-chain variable fragment (scFv) of the anti-GCN4 antibody fused to a VP64, creating a scaffold able to recruit up to 25 copies of VP64. An up to 50-fold increase in gene expression has been achieved when targeting the *CXCR4* (C-X-C Motif Chemokine Receptor 4) gene, showing a much higher efficiency than the classical dCas9-VP64 (up to 2-fold increase) (Tanenbaum et al., 2014). This strategy has also been adapted for epigenetic modulation as well by fusion with different effector proteins, a strategy we will describe in a further section of this review.

##### 2.1.1.3 dCas9-SAM system

The Synergistic Activation Mediator (SAM) system requires the conventional dCas9-VP64 alongside engineered sgRNAs, to incorporate aptamers for MS2 proteins (Konermann et al., 2014). The system leads to the assembly of a synthetic activation complex, adapted from the natural transcriptional machinery and that consists of three proteins: the MS2, p65 and the Heat Shock Transcriptions Factor 1 (HSF1), or the MS2-p65-HSF1 (MPH) complex. Briefly, binding to the modified sgRNA occurs via MS2, and transcription is facilitated via the p65 and HSF1 domains (Konermann et al., 2014) (Figure 3D)

All these systems promote gene expression at a higher level than the original dCas9-VP64. Nonetheless, the SAM system consistently shows the best activation levels of all, when compared to the SunTag and VPR (Chavez et al., 2016; Becirovic, 2022). Activation efficiency varies according to the basal expression of the target gene, meaning that higher basal levels tend to lead to a reduced induction. However, the SAM system induction was observed to be up to five-fold higher than the SunTag or VPR, in the same gene panel (Chavez et al., 2016). An advantage of using the VPR system is its simplicity, as it requires only a simple fusion protein, facilitating delivery. On the other hand, the SunTag disadvantage lies in its antibody chain scaffold, as it can be inconsistently expressed in cells. Lastly, for the SAM system, its efficiency decreases in multiplex approaches when compared to its use with a single sgRNA (Chavez et al., 2016). In all cases, a good sgRNA design is a critical requirement for gene expression promotion (Xu et al., 2015).

The advancement of improved systems and the combination of elements from both SunTag and SAM systems has led to the development of the dCas9-SunTag-p65-HSF1 (SPH) (Zhou et al., 2018). In this system, the VP64 used in the SunTag system is replaced with the p65-HSF1 (Figure 3E), from the SAM system. In terms of platform efficiency, SPH platform is able to promote the highest increase in gene activation when compared to other systems (Zhou et al., 2018).

##### 2.1.1.4 Systems for epigenetic modulation

As previously mentioned, an alternative strategy for gene expression activation is the modulation of the chromatin landscape in the target locus. In line with this, various systems have been developed for epigenetic editing, based on the combination of known modulators like DNA or histone methylases and demethylases. For instance, combination of a dCas9 with the Ten-Eleven translocation dioxygenase 1 (TET1) catalytic domain (TET1-CD) results in the targeting and demethylation of promoter regions, consequently leading to gene upregulation (Choudhury et al., 2016). The same effector has been used in additional studies to modulate CpG island methylation state and increase gene expression (Liu et al., 2016; Morita et al., 2016; Xu X. et al., 2016). Fusing the TET1 to an adapted SunTag has been applied both *in vitro* and *in vivo* in a mouse model*,* inducing a 1.7- to 50-fold increase in gene expression (Morita et al., 2016). Additionally, a two-component system consisting of a fusion protein between dCas9-TET1-CD and MS2 coating proteins, and a modified sgRNA with MS2 elements, has also been used to target hypermethylated genes. The promoter regions of several genes have been targeted (either in single- or multiplex), demethylated and transcription induced, leading to an upregulation (Xu X. et al., 2016). These studies demonstrate the possibility to target hypermethylated regions and effectively modulate their methylation levels. Considering how DNA hypermethylation can, in some cases, lead to cancer initiation and progression, these systems can be applied to develop novel therapeutic strategies in the oncological field.

In turn, H3K27ac (acetylation of lysine 27 of the H3 histone protein) is another epigenetic mark usually correlated with gene upregulation. As so, dCas9 has been fused with the acetyltransferase catalytic domain of the human E1A-associated protein p300, in order to manipulate the acetylation state of target gene promoters and enhancers, increasing or activating gene expression (Hilton et al., 2015). However, contrary to other systems, activating efficiency did not increase by using multiple sgRNAs (Hilton et al., 2015).

An important consideration for the efficiency of previously reported fusion tools is the epigenetic state of the targeted region. Initial reports supported a direct correlation between target’s epigenetic status and dCas9 binding (Wu et al., 2014; Horlbeck et al., 2016). In hypermethylated regions, with decreased chromatin accessibility, dCas9 binding was shown to be hampered (Horlbeck et al., 2016). However, more recently, it has been shown that this association between chromatin structure and Cas9 binding efficiency is dependent on intracellular Cas9 levels and exposure time (Kallimasioti-Pazi et al., 2018). This means that higher Cas9 concentrations lead to increased efficiency both in terms of heterochromatin and euchromatin modulation.

CRISPRa technology has proved to be advantageous due to target gene size independence and multiplex potential. The possibility of off-target effects, compared to conventional CRISPR scissors, is much smaller as the target sequence must be located close or within regulatory regions (such as promoters or enhancers) (Zhang et al., 2018; Becirovic, 2022). Other significant advantages of CRISPRa are the capacity to regulate all possible gene isoforms or in contrast, to enable targeted activation of specific isoforms. This is particularly valuable when studying rare diseases characterized by tissue-specific isoform expression (Terkelsen et al., 2024). Nonetheless, there are limitations to its application. For instance, no permanent genome modifications are achieved when using CRISPRa. Thus, long-term system activity requires the use of viral-based delivery systems or multiple administrations. Additional challenges include the risk of an immune response against the bacterial system (Crudele and Chamberlain, 2018; Charlesworth et al., 2019) and, due to vectors’ large size, the packaging capacity of some delivery systems which can be circumvented with separate protein delivery (Liu et al., 2017; Vora et al., 2018). Nevertheless, the lack of permanent changes to the DNA sequence can be beneficial if the goal is to prevent passing the modification down to progeny. Therefore, a thorough risk/benefit assessment is necessary depending on the intended application.

#### 2.1.2 CRISPR inhibition/interference systems

**FIGURE 4:**
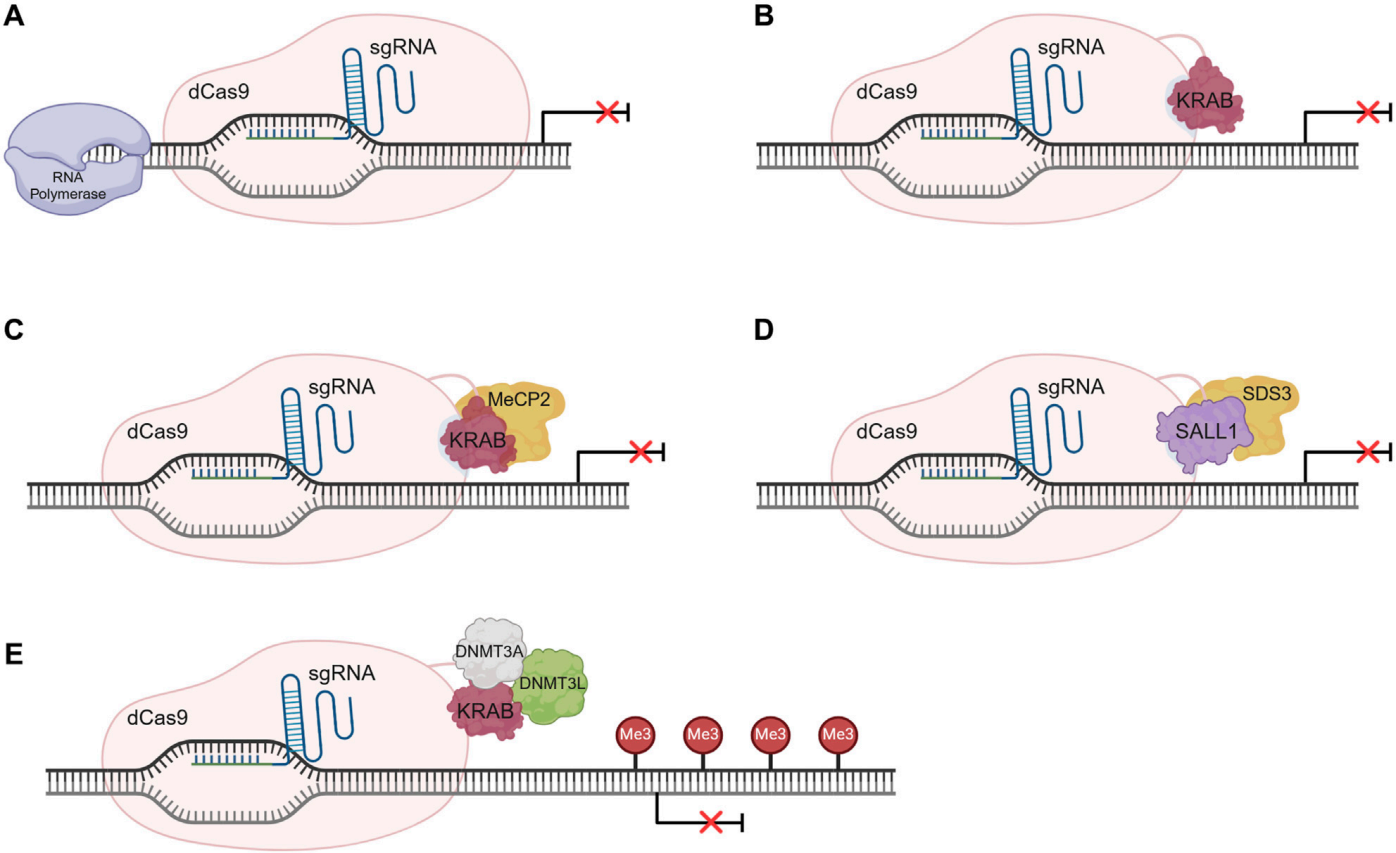
Overview of CRISPR interference (CRISPRi) systems’ mechanism of action. **(A)** Gene repression exclusively recurring to dCas9-sgRNA complex. The complex sterically blocks the action of the RNA polymerase impeding gene transcription initiation. **(B)** dCas9-KRAB-sgRNA system mechanism of action. Gene expression inhibition is promoted by the transcription repressor KRAB, fused to the dCas9, by chromatin conformation modulation. **(C)** dCas9-KRAB-MeCP2 system for gene repression. The bipartite fusion protein can interact with epigenetic regulators through the MeCP2 domain promoting gene silencing. **(D)** Gene silencing using the dCas9-SALL1-SDS3 system. The bipartite fusion protein can interact with the histone deacetylases, inducing epigenetic architecture alterations that lead to gene silencing. **(E)** Permanent gene repression by CRISPRoff using the dCas9-KRAB-DNMT3A-DNMT3L fusion protein. The induced DNA methylation results in gene repression and is stably inherited through cell division, rendering the continuous presence of the CRISPR system unnecessary. Illustration created using BioRender.

Transcriptional repression can be achieved using a CRISPR inhibition or interference (CRISPRi) approach. CRISPRi is able to downregulate native gene expression without degrading mRNA transcripts, unlike short interfering RNA (siRNA) approaches. Figure 4 provides an overview of some CRISPRi systems.

The simpler system relies on the action of a dCas9-sgRNA complex to the target gene’s regulatory region, sterically blocking RNA polymerase activity, and halting transcription initiation or elongation (Figure 4A), depending on the target locus (Qi et al., 2013). In mammalian cells, a reduced silencing efficiency is observed, mostly due to the complexity of transcriptional regulatory pathways which involve genetic and epigenetic factors. Additionally, repression can be achieved using a fusion protein constituted of a dCas9 and a transcriptional repressor like the Kruppel-associated box (KRAB) domain–Figure 4B. KRAB interacts with the scaffold protein KAP1, which recruits histone modification proteins, leading to alterations in heterochromatin conformation and consequently to gene repression, either at promoter or enhancer sites (Qi et al., 2013; Gao et al., 2014; Thakore et al., 2015). An alternative strategy is the promotion of *de novo* methylation at regulatory regions using a dCas9 fused to DNA methyltransferase (DNMT) 3, which can lead to gene repression (Liu et al., 2016).

More complex fusion proteins, encompassing more than one effector, can also be used for gene repression (Figure 4C). An example is the fusion of KRAB domain to the Methyl-CpG Binding Protein 2 (MeCP2) that creates the dCas9-KRAB-MeCP2 bipartite construct. This system is more efficient than the dCas9-KRAB, as, in addition to the KRAB mechanism of action, the MeCP2 domain can interact with regulators such as DNMT1 and the SIN3A-histone deacetylase corepressor complex, further promoting epigenetic silencing (Yeo et al., 2018). Nonetheless, additional work is needed to increase system specificity. Recent strategies use repressor domains like the Sal-like protein 1 (SALL1) and Sin3a corepressor complex component (SDS3), in a bipartite construct named dCas9-SALL1-SDS3 (Mills et al., 2022). (Figure 4D) This next-generation system induces gene silencing to a greater extent than simpler systems, given its ability to interact with functional effectors like histone deacetylases (HDAC), changing the epigenetic landscape.

As with CRISPRa, unless constitutively expressed, CRISPRi-induced gene expression regulation is transient. Lombardo and colleagues were able to develop a permanent and inheritable system for gene silencing in somatic cells, using different epigenome editing platforms, including CRISPR-dCas9 (Amabile et al., 2016). Briefly, three individual fusion proteins were engineered and delivered to the cells. dCas9 was separately fused with KRAB, DNMT3A or DNMT3L domains, and all fusion forms were then delivered to the cells altogether and targeted to the promoter region of β2-microglobulin (*B2M*) gene, to modify its epigenetic state. This strategy led to a long-term and efficient repression of the target gene, in K-562 cells. This work also demonstrated the possibility to target and silence multiple genes, simultaneously. More recently, Nuñez and colleagues (Nuñez et al., 2021) developed a more complex fusion protein consisting of dCas9 fused to all the above mentioned protein domains concurrently–KRAB, DNMT3A (D3A), and DNMT3L (D3L) – creating a strategy designated CRISPRoff (Figure 4E). The recruitment of histone deacetylases, methyltransferases, and heterochromatin protein 1 leads to DNA methylation and the acquisition of an inactive heterochromatin state, altering the epigenetic marks of the genomic locus. This newly formed repressive epigenetic landscape is heritable and stable, being maintained throughout cell division. Nonetheless, the alterations can be reversed using targeted DNA demethylation. CRISPRoff is more versatile than other CRISPRi systems, as it can be used to silence regions with no CpG islands and has been proved to work with dCas12a (Nuñez et al., 2021).

## 3 CRISPR-based preclinical studies targeting IVD degeneration

As abovementioned, the versatility of CRISPR technology can be applied to the field of IVD regeneration either alone or in combination with other biological approaches. So far, regenerative strategies for the disc have been mostly focused on cell-based therapies, biomaterial-based approaches and genetic engineering. MSCs, for instance, have been widely used, due to their self-renewal and differentiation potential. They can either be directly injected into the IVD (Centeno et al., 2017; Noriega et al., 2017) or differentiated, *in vitro,* into NP-like cells, prior to its use (Stoyanov et al., 2011; Clarke et al., 2014). Additionally, MSCs secretome stimulates cell proliferation and ECM synthesis while reducing the secretion of pro-inflammatory factors (Shim et al., 2016; Ferreira et al., 2018). This immunomodulatory effect of MSCs can be beneficial to balance the degenerated microenvironment, reducing both inflammation and ECM degradation. However, cell-based approaches face several challenges regarding cell homing and survival due to the harsh IVD environment, with high osmotic pressures, hypoxia and oxidative stress (Croft et al., 2021). An additional drawback is cell leakage, which has been associated with ectopic bone formation (Vadalà et al., 2012). Moreover, multiple injections are anticipated to be required for MSC-derived secretome administration to achieve a sustained effect.

Given the bottlenecks of cell-based therapies, CRISPR constitutes a promising alternative to enhance therapeutic effectiveness by improving cell survival, promoting ECM deposition, and reducing inflammation. At the same time CRISPR offers a therapeutic approach to modulate the catabolic and pro-inflammatory environment, regulate pain-sensing pathways, enhance ECM synthesis and growth factors production, and target polymorphisms associated with DDD. Most studies using CRISPR focus on better understanding cell-ECM interaction, uncovering mechanisms of the degenerative process and pain modulation, and revealing novel therapeutic targets for IVD regeneration. A brief overview of IVD studies applying the CRISPR toolbox are summarized in Table 2.

**TABLE 2:** Intervertebral Disc studies using CRISPR technology. White rows list studies regarding cell-ECM interactions. Gray rows list studies associated with inflammation and low back pain.

CRISPR system	Target	Delivery into host	Model of the Study	Main Outcomes	Challenges and Opportunities	References
CRISPR-Cas9 Sgp	*Chsy3*	Transfection | Semi-cloning	Chsy3^−/−^ Mouse Model	↓ Aggrecan, NP cells, disc heigh and hydration	*In vivo* IVD degeneration model to uncover novel targets. Translation to humans is an issue	Wei et al. (2020)
dCas9-KRAB Sgp	*Parkin*	Transfection	Rat Nucleus Pulposus cells	Loss of Parkin protective role; NP cell apoptosis	New DDD target needs to be validated *in vivo* and in human cells.	Lan et al. (2021)
dCas9-VPR Sgp	*Vdr*	Transfection	Rat Nucleus Pulposus cells	Oxidative damage improvement and ↓ NP cell apoptosis	Novel therapeutic target in DDD but needs to be complemented with *in vivo* studies and human cells	Lan et al. (2022)
CRISPR-Cas9 Sgp	*Ctnnb1*	AAV transduction	*In vivo*: Mouse model of DDD	Injury-induced disc degeneration amelioration	Therapeutic target for DDD but *in vivo* model translation to human is an issue	Fan et al. (2022)
CRISPR-Cas9 Sgp	*Nudt21*	LV and Hydrogel-based	DDD Mouse model and Mouse NP cells	NP degeneration improvement; ↑ Cell proliferation	Human validation needed; Novel delivery system for IVD and target for DDD.	Yu et al. (2024)
dCas9-VPR Mtp	*ACAN, COL12A1, ZNF865*	LV transduction	Human adipose- derived stem cells	↑ Cartilaginous tissue deposition; ↑ Mechanical properties	Human validation needed. Enhance cell-based therapies’ outcomes	Levis et al. (2025)
dCAS-KRAB Sgp	*Akap150*	LV transduction	Rat Dorsal Root Ganglia neurons	Ø DDD-induced DRG-elevated neuron activity	Possible neuromodulator in other tissues. Novel LBP therapeutic target. Human validation needed	Stover et al. (2017)
dCAS-KRAB Sgp and Mtp	*Tnfr1, IL1R1, IL6st*	LV transduction	Rat Dorsal Root Ganglia neurons	Role of IL-6, TNF-α, and IL-1β in pain sensitization in degeneration	Multiplex approaches within the signaling pathways players for better results. Human validation needed	Stover et al. (2019)
dCas9-KRAB Sgp	*TNFR1* and *IL1R1*	LV transduction	Human NP cells	Inhibition of TNFα–mediated inflammation, cell death, and catabolism	System improvement required for *IL1R1* editing. Validation *in vivo* needed. Protective effects validated in human cells	Farhang et al. (2019)
dCAS-KRAB Sgp and Mtp	*IL-6*th*, Asic3, Trap1, Piezo1*, *Piezo2*	LV transduction	Rat Dorsal Root Ganglia neurons	Ø DDD-induced mechanical sensitization of nociceptive neurons	Neuromodulation strategy for LBP treatment. Human and *in vivo* validation required	Stover et al. (2023)
CRISPR-Cas9 Sgp	*Ntn1*	AAV transduction	*In vivo:* Rat model of DDD	↓ Nerve innervation and angiogenesis	Novel therapeutic approach for LBP. Human validation needed	Zheng et al. (2023)

Sgp: Single plex/Mtp: Multiplex/LV: Lentiviral/RNP: Ribonucleoprotein/Ø: Ablation/HAC: human articular chondrocytes.

### 3.1 Cell-ECM modulation studies

NP cell deficiency is a characteristic of IVD degeneration and oxidative stress is important in NP cell apoptosis. To better understand this process, researchers have transfected primary rat NP cells with the CRISPR/dCas9-KRAB system to downregulate *Prkn* (Parkin RBR E3 Ubiquitin Protein Ligase). Loss of Parkin was translated into increased NP cell apoptosis and autophagy inhibition, which further contributed to the progression of IVD degeneration (Lan et al., 2021). Considering the protective role of autophagy under oxidative stress, against cell apoptosis, the same group used CRISPRa technology to target autophagy regulators. Using the abovementioned strategy, but with VPR system, Lan and colleagues focused on overexpressing vitamin D receptor (VDR), which is usually under expressed in degenerated discs. Increased levels of VDR promoted mitophagy and prevented apoptosis, in H_2_O_2_ treated rat primary NP cells (Lan et al., 2022). Altogether, the authors suggest a future therapeutic approach for IVD degeneration, either by targeting Parkin and/or increasing VDR expression in the disc. These reports show that engineering NP cells *ex vivo* is feasible and could open the door for future studies targeting known polymorphisms associated with DDD (Mayer et al., 2013).

CRISPR/Cas9 has been used to understand the role of β-catenin in the degenerative process and to explore its potential as a therapeutic target for IVD degeneration. β-catenin is known to be involved in IVD development and metabolism through the Wnt/β-catenin pathway and its levels are upregulated in degenerated discs, suggesting an association with IVD pathology (Kondo et al., 2011; Xu H. et al., 2016). To better understand β-catenin function in DDD, Fan and colleagues targeted the *Ctnnb1* (Catenin Beta 1) gene using CRISPR/Cas9 vectors packaged into an adeno-associated virus (AAV) (Fan et al., 2022). The system was first tested in rodent CD45^−^ bone marrow stromal cells, leading to an *in vitro* ablation of β-catenin, prior to viral-mediated delivery into a mouse model of disc degeneration. This *in vivo* study showed that β-catenin depletion led to a better preservation of IVD structure, a reduced shortfall of notochordal cells and a decrease in MMP13 and ADAMTS5 (Disintegrin and metalloproteinase with thrombospondin motifs) production (Fan et al., 2022).

In another study, researchers focused on understanding the impact of chondroitin sulfate loss during IVD degeneration and the role of chondroitin synthase 3 (*Chsy3*) in its biosynthesis (Wei et al., 2020). Chondroitin sulfate is the most abundant glycosaminoglycan (GAG) present in the NP and has a pivotal role in disc hydration, thus being critical for the maintenance of IVD function (Collin et al., 2017). A CRISPR/Cas9 system was used to create a *Chsy3* knock-out (KO) mice model, to investigate the molecular function of Chsy3. An accelerated degenerative process was observed in *Chsy3* ^−/−^ mice, when compared to wild-type, due to the depletion of aggrecan, loss of NP cells, reduced disc heigh and tissue dehydration (Wei et al., 2020). *In vitro*, when using primary NP cells from these KO mice, an upregulation of ADAMTS4/5 and MMP2/13, as well as a downregulation of the Hippo signaling pathway through Yap1 (Yes-associated protein 1) (Wei et al., 2020) was observed. This new *Chsy3* ^−/−^ mice model of IVD degeneration, generated using CRISPR technology, will be key to uncover novel potential targets and therapies.

Considering the role of MMPs in healthy and degenerated tissues, decreasing their levels without completely shutting them down is key to modulate matrix turnover. An important player in the natural ECM homeostasis is MMP13, as it can degrade collagen type II at a much higher rate than other proteases (Mitchell et al., 1996). However, considering that type II collagen is the most abundant molecule in the NP, and that its levels decrease with degeneration, MMP13 can be a good therapeutic target for DDD. Seidl and colleagues developed a CRISPR/Cas9 system targeting *MMP13* to promote downregulation of the corresponding enzyme in human articular chondrocytes. However, by not selecting biallelically edited cells they were able to obtain a reduction of MMP13 levels without complete ablation (Seidl et al., 2019). In 3D spheroid cultures, engineered cells showed reduced MMP13 expression and increased levels of collagen type II, which the authors refer to as a chondroprotective effect (Seidl et al., 2019). This strategy can be used in combination with cell-based therapies using chondrocytes, to increase their efficiency while maintaining sufficient MMP13 expression close to biological levels found during natural ECM turnover. Despite being feasible to use it alone as genetic therapy, some adaptations to the system are required. Considering that in this work, Cas9 was delivered to the cells as a ribonucleoprotein, *in vivo* delivery is hindered due to challenges in its encapsulation, as a result of its size and charge. Thus, shifting to a viral-based delivery system, using an AAVs or lentivirus might be necessary for translation. Nonetheless, allelic ablation *in vivo* is more complex and still in its infancy.

A recent study by Levis *et al.* explored the potential of CRISPRa in addressing IVD degeneration by manipulating matrix components. The research focused on ZNF865, a zinc finger protein crucial for cell cycle progression, DNA replication, and protein processing. By employing CRISPRa to upregulate ZNF865 in human adipose-derived stem cells (hASCs), researchers observed a significant increase in the synthesis of key structural constituents such as GAGs and collagen II, essential for maintaining architectural integrity and biomechanical properties of the IVD. Furthermore, ZNF865 upregulation activated TGF-β (Transforming growth factor beta) signaling pathway, an essential regulator of ECM synthesis. In addition, ZNF865 overexpression enhanced SOX9 (SRY-Box Transcription Factor 9), a master controller of chondrogenesis, likely contributing to the observed improvement in cartilage-specific ECM production. For *in vivo* validation, a rat tail puncture model of IVD degeneration was used. Injection of ZNF865-overexpressing hASCs resulted in a significant increase in disc height and proteoglycan content, as well as structural improvement in the treated groups. These findings suggest that CRISPRa-mediated upregulation of ZNF865 could be a promising approach for IVD regeneration, offering a potential therapeutic strategy to address disc degeneration through enhanced ECM production and maintenance of disc structure (Levis et al., 2025).

Yu and colleagues proposed an innovative approach for IVD regeneration combining CRISPR technology with advanced materials (Yu et al., 2024). They developed a novel spherical Gelatin Methacryloyl (GelMA)/Hyaluronic Acid Methacryloyl (HAMA) hydrogel encapsulating APET × 2 polypeptide and a CRISPR-Cas9 system targeting *Nudt21* (Nudix Hydrolase 21). This gene encodes CFIm25 (Component of the cleavage factor Im*)*, a protein significantly upregulated in degenerated NP tissue that correlates with disease progression. Using an *in vivo* mouse model of IVD degeneration, CFIm25 downregulation promoted NP cell proliferation and migration, and increased disc height, reducing IVD degeneration score. Furthermore, this study’s findings revealed that reducing CFIm25 expression through this CRISPR-based approach resulted in the inhibition of inflammatory factors (IL-6, inducible nitric oxide synthase - iNOS, IL-1β, TNF-α) by modulating p38/NF-κB signaling pathway. Expression of crucial ECM components (collagen II and aggrecan) was also increased, while COX2 (Cyclooxygenase-2) and MMP3 levels were suppressed (Yu et al., 2024). This approach offers a promising direction for developing more effective and tailored therapies for IVD regeneration.

The role of other structural IVD components has also been uncovered in the last years. Proteomic analysis of the NP ECM has shown how matrissome changes throughout development, in bovine and human samples (Caldeira et al., 2017; Rajasekaran et al., 2020; 2023). These studies have identified new targets for either activation or downregulation, through CRISPR-based methodologies. Such high-throughput analysis has opened the door to the development of new approaches to recapitulate a healthy microenvironment, promote cell survival and/or modulate ECM turnover. Strategies involving CRISPR for ECM tailoring are starting to emerge (Zhai et al., 2023), thus an organic growth of this approach and translation to IVD regeneration studies is expected. Additionally, considering that some matrix components are hard to obtain, even recurring to recombinant proteins, CRISPR-based cell lines able to produce such components in large scale could help to explore a new range of products to either functionalize biomaterials or use as coatings.

### 3.2 CRISPR strategies for inflammation and discogenic pain

Low back pain can stem from various factors such as muscle strain, joint dysfunction, spinal stenosis, or nerve compression. However, discogenic pain due to IVD degeneration remains a significant contributor. One of the hallmarks of the degenerative state is the presence of an inflammatory environment. Thus, a pro-regenerative strategy may focus on microenvironment modulation by targeting cytokines and signaling pathways, either *in vitro*, *in vivo* or *ex vivo*. By controlling these pathways one can uncover which candidates provide a protective effect against disc degeneration. In human degenerated NP cells, Farhang and colleagues used CRISPR/dCas9-KRAB, to downregulate the expression of TNFR1 (Tumor Necrosis Factor Receptor-1) or IL1R1 (Interleukin 1 Receptor Type 1*)*, which are receptors for TNF-α and IL-1β, respectively (Farhang et al., 2019). These two cytokines constitute valuable therapeutic targets as they play a pivotal role in DDD, promoting apoptosis and ECM degradation, consequently propagating a catabolic microenvironment. Despite being able to downregulate both *TNFR1* and *IL1R1*, editing efficiency was higher and more consistent in TNFR1-edited cells (>85%). Focusing on these cells, post TNF-α supplementation, there was a significant decrease in NF-κB induction, which indicates TNFR1 signaling inhibition. Additionally, there was a reduction of both cell apoptosis and catabolic gene expression. TNFR1-edited cells showed a protective effect by maintaining aggrecan production and decreasing MMP13 levels (Farhang et al., 2019). Altogether, this strategy can be used to increase the efficiency of cell-based therapies by improving cell survival through modulation of the IVD inflammatory environment. Alternatively, it can constitute a genetic therapy to ameliorate DDD, per se.

The specific mechanism of discogenic pain onset is yet to be uncovered, thus CRISPR potential can be harnessed to better understand this condition’s etiology and progression, as well as to uncover novel therapeutic targets and develop advanced treatments. In 2022, Zheng and colleagues delved into the mechanism of discogenic pain by confirming the role of Netrin-1, a factor involved in axonal growth, in this condition (Zheng et al., 2023). In this loss of function study, the authors used a CRISPR-Cas9 system, targeting the *Ntn1* (Netrin 1) gene to deplete Netrin-1 *in vivo*, by AAV injection, in a rat model of disc degeneration. Nectrin-1 reduction led to pain relief in the animals. Thus, it might constitute a promising target to tackle LBP. Additional candidates to consider are cytokines and their receptors, considering their effect in the progression of IVD degeneration and in the onset of pain (Risbud and Shapiro, 2014; Johnson et al., 2015). Nonetheless, more work is needed to fully unveil their specific role and therapeutic potential.

The inflammatory microenvironment of the degenerated IVD is associated with dorsal root ganglion (DRGs) neuronal activation and pain sensation (Risbud and Shapiro, 2014). It is considered that the degenerative environment leads to the sensitization of nociceptive neurons, that activate in response to a stimulus considered non-painful when in healthy patients. Bowles’ group has been focused on understanding the role of inflammatory cytokines and their signaling pathways in altered neuron nociception, in the context of IVD degeneration. To achieve this, it is crucial to consider the interplay between the alterations that occur in IVD components and in sensory neurons. Using a CRISPR epigenome editing strategy to modulate rat DRG neuronal activity, Stover and colleagues developed *in vitro* models to study the effect of IVD degenerative microenvironments in nociception (Stover et al., 2017; Stover et al., 2019; Stover et al., 2023). Initially, they showed that conditioned media from human degenerated IVD samples led to an increase in DRG neuronal activity *in vitro*, in response to thermal stimuli. This stimulation was induced by IL-6 and could be modulated by A-kinase anchoring proteins (AKAP) (Stover et al., 2017). Thus, to influence neuronal response, Stover and colleagues used a dCas9-KRAB system to target the AKAP150 promoter region by lentiviral transduction of rat DRG neurons *in vitro*. Downregulation of AKAP150 eliminated nociceptive neuronal activity induced by the degenerated IVD but maintained physiological activation to calcium (Stover et al., 2017). This work showed the role of the IVD degenerative environment, in particular of pro-inflammatory cytokines, on the sensitization of nociceptive neurons, shedding light on LBP mechanism.

Further work was developed by the Bowles group, using the same CRISPR epigenome editing strategy, to better understand the intervenients in neuronal activation. Three cytokines’ receptors - *IL-6*th, *IL-1R1* and *TNFR1* – were targeted, using a single or multiplex approach, to identify enhanced neuronal activity mediators. Individual depletion of each of these cytokines led to a reduction of neuronal activity induced by pathological IVD tissue exposure. However, multiplex editing completely abolished neuronal activity triggered by the exposure to human degenerative discs (Stover et al., 2019). This work provided new insights for clinical translation, as most research is focused on the discrete role of different cytokines in degeneration. It is therefore necessary to consider the combined interplay between inflammatory mediators to achieve better clinical outcomes when developing long-term solutions for discogenic pain.

In parallel with inflammation, mechanical loading further contributes to the progression of IVD degeneration and LBP. However, the pathways involved in this mechano-inflammatory interaction are yet to be understood. To narrow this knowledge gap, Cambria and colleagues looked into the role of the mechanosensitive ion channel TRPV4 (Transient Receptor Potential Cation Channel Subfamily V Member 4) (Cambria et al., 2020). Using a CRISPR-Cas9 system they were able to transduce human AF cells with lentivirus and knock-out *TRPV4*. Depletion of TRPV4 reversed the upregulation of IL-8 and IL-6, which is usually induced by stretching stimuli (Cambria et al., 2020). Given that these pro-inflammatory cytokines are clinically relevant in the context of IVD degeneration and discogenic pain, TRPV4 seems to be an interesting target for LBP therapeutics. Additional work on the disc mechano-inflammatory interaction was developed by Bowles’ group. Stover and colleagues identified three ion channel mediators–*Trpa1* (Transient Receptor Potential Cation Channel Subfamily A Member 1), *Asic3* (Acid Sensing Ion Channel Subunit 3) and *Piezo2* (Piezo Type Mechanosensitive Ion Channel Component 2) – involved in degeneration induced nociception in the IVD (Stover et al., 2023). DRG neurons were epigenetically edited by lentiviral transduction with a dCas9-KRAB system targeting *Trpa1*, *Asic3* and *Piezo2* either individually or through a multiplex approach. Singleplex editing led to a reduction of nociception neuronal activation, in response to mechanical stimuli, in a degenerative environment. Nonetheless, the multiplex approach, in cyclic strain conditions, showed not only absent neuronal activation in response to mechanical stimuli, induced by disc degeneration, but also allowed to uncover the combined contribution of TRPA1, ASIC3 and PIEZO2 in this mechanosensing pathway (Stover et al., 2023). Altogether, these works unveiled the potential of CRISPR for neuromodulation, increasing the range of strategies to tackle LBP.

## 4 Discussion and conclusion

The CRISPR toolbox offers unprecedented potential for applications across different research fields and despite having been available for over a decade, its recent growth in the IVD field is noteworthy. Harnessing this technology to narrow current IVD biology knowledge gaps, through functional studies or IVD disease modelling is crucial to develop effective therapies for disorders. Current literature is focused on uncovering targets in the ECM, pain sensing and/or inflammatory pathways. As we learn more about the IVD tissue in health and disease, the knowledge gathered could be used to recapitulate healthy conditions in degenerated discs and potentially develop novel disc regeneration approaches. CRISPR methodology is versatile enough to allow this type of strategy, as it can be used to reactivate silenced genes or just boost the expression of factors that decrease in a disease context. Understanding IVD development and not only healthy adult tissue is also important, as degeneration occurs naturally with age and healthy tissue can already bear a few non-pathological alterations. Harnessing the potential of CRISPR to develop novel models of IVD degeneration is crucial to overcome the limitations of current animal models. It is known that no model is flawless, but the CRISPR system could help narrow the observed differences, to facilitate translation.

Using CRISPR to target SASP could also be a promising approach to mitigate IVD degeneration. Disrupting BCL-2 (B-cell lymphoma 2) or HSP90 (Heat Shock Protein 90) through CRISPR editing to eliminate senescent cells as an alternative to senolytic drugs (Kamali et al., 2021; Liu et al., 2024), or suppressing BRD4 (Bromodomain-containing protein 4) could eliminate SASP-driven inflammation and ECM degradation, potentially halting the progression of the disease and restoring disc homeostasis.

Studies on the mediators of IVD degeneration have also unraveled the importance of targeting multiple factors simultaneously. Nonetheless, the number of studies using multiplex approaches in the IVD field is still scarce and research is mainly performed by a single lab. In the future multiplexing should be further explored as it has been shown to improve treatment efficacy with promising *in vivo* results.

CRISPRa has been used to boost the therapeutic effect of extracellular vesicles (EVs) from edited MSCs. Martinez-Zalbidea and colleagues have established a CRISPRa system for TSG-6 overexpression in MSCs, by lentiviral transduction with dCas9-SAM system (Martinez-Zalbidea et al., 2025). IL-1β-stimulated human IVD cells treated with EVs from these engineered cells showed a decrease in IL-8 and COX2 pro-inflammatory cytokines. Cell-based, cell-free products are a nice therapeutical alternative, as they can overcome some regulatory challenges for clinical translation.

Recently, the concept of recapitulating the fetal environment to regenerate tissues has been on the rise (Aztekin, 2024; Viragova et al., 2024). Despite not being a novel strategy (Jensen et al., 2005), combination with gene editing techniques could prove quite successful. Considering how the IVD regenerative potential is confined to its fetal stages, disc regeneration could be promoted by recapitulating fetal cues (Fiordalisi et al., 2022). This could be pursued with the aid of CRISPRa and CRISPRi systems.

The recent approval of CRISPR-Cas9 as a treatment for sickle cell disease and β*-*thalassemia by regulatory agencies, will certainly foster further developments in distinct fields. Noteworthy, despite the huge panoply of registered clinical trials using CRISPR systems, none is yet exploring CRISPR activating or inhibition technology, nor are they focusing on intervertebral disc disorders.

The ultimate groundbreaking technology for genome editing that is still to be applied in the IVD field is base editing. This method can be used to generate specific point mutations, either in genomic DNA or RNA, with no need for a donor template nor for the generation of DSB (Komor et al., 2016). Thus, its application is anticipated for correcting polymorphisms described in the context of IVD degeneration.

As with all technologies, there are limitations associated with the use of CRISPR *in vivo*, mainly regarding delivery strategies, off-target effects and ethical issues. Delivery of the components *in vivo* has been performed using viral vectors, specifically lentiviral-based systems. To continue doing so, studies need to address possible immunogenicity and safety concerns from regulatory entities. An alternative involves AAV systems, which are less immunogenic than lentiviral vectors, and have been used for gene delivery in several clinical trials aimed at hindering degenerative disc disease progression. Nonetheless, the use of AAV systems also comes with limitations - their small genome size impairs the cloning of large genes (Kabadi et al., 2024).

At the same time, delivery strategies need to consider the challenging anatomical organization and localization of the disc. An injection is required to reach the target tissue, specifically the nucleus pulposus, for which it is crucial to use very thin needles to minimize damage and to carefully consider the volumes to be injected due to the high level of degeneration. The harsh microenvironment of the IVD poses additional challenges for CRISPR efficacy, such as low oxygen levels, acidic pH, and limited nutrient availability can impair cellular uptake and gene-editing efficiency. Strategies to overcome these barriers include engineering CRISPR components to function optimally under these conditions or co-delivering supportive molecules like oxygen carriers or buffering agents, (Krupkova et al., 2018; Tsuchida et al., 2024). It is also necessary to account for osmotic pressure and the risk of possible leakage, which could reduce efficiency and lead to potential unintended outcomes.

Concerning undesired effects and associated safety issues related to the delivery system, they can be reduced by using viral vectors with increased specificity to the target cell or tissue of interest. This has already been tested for cartilage, for instance. Thus, screening for or engineering IVD-specific viral vectors is a topic worth exploring for the development of future therapies (Yoon et al., 2021). *In vitro*, for instance, AAV-2 and AAV-6 have shown the best tropism in human NP cells (Mern and Thomé, 2015)*,* whereas in rabbits AAV-6 performed better (Kim et al., 2022).

In turn, non-viral delivery methods for the CRISPR system constitute safer alternatives to viral vectors, addressing immunogenicity and scalability (Sinclair et al., 2023). Examples include lipid nanoparticles (LNPs) (Chen et al., 2023), polymer-based systems (Zhang et al., 2020; Sinclair et al., 2023), hybrid platforms (Gameiro et al., 2024; Lee et al., 2025), and physical methods like electroporation, despite invasive and hard to scale (Sinclair et al., 2023). Additional challenges include nanoparticle stability and cellular uptake in the harsh IVD environment (Tang et al., 2019).

Moreover, off-targets of the CRISPR system itself can be mitigated by enhancing sgRNA precision through the use of *in silico* predictive tools for designing and selecting high-scoring sgRNAs with fewer predicted unspecific binding sites. Among these tools is CRISOT, developed by Chen and colleagues, which provides a genome-wide CRISPR off-target prediction and optimization platform (Chen et al., 2023).

Despite these advancements, efficient gene editing may also be hampered by IVD non-dividing cells. CRISPR systems often rely on cellular machinery which is more active during cell division (e.g., homologous recombination), limiting its effectiveness in quiescent or senescent cells, commonly found in degenerated discs. Other approaches such as base or prime editing may offer alternative solutions by enabling precise alterations without relying on DNA repair pathways typically active during cell division. However, these technologies are still under development and require further optimization before clinical application (Li et al., 2018; Sioson et al., 2021; Subica, 2023).

Altogether, the safety concerns associated with the use of genome editing technologies raise additional ethical discussions regarding its use in a clinical setting. Moreover, germline cells editing has sparked an open debate, as using CRISPR for therapeutic approaches can open a precedent for non-therapeutic editing. Equitable access is also a matter of concern as the high costs of these therapies could exacerbate healthcare disparities if they are only accessible to certain populations or regions. Ensuring affordability and fairness in distribution will be key as these therapies advance towards clinical implementation (D’Souza et al., 2023; Subica, 2023). The balance between risks and benefits remains a hot topic among researchers and bioethicists. This ongoing dialogue underscores the potential of CRISPR and should continue to be fostered as we gain a deeper understanding of the technology and its limitations.

The emerging trend for IVD studies using genome engineering has provided great insights and promising results regarding the identification of novel therapeutic targets for disc degeneration and discogenic pain. In the years to come, efforts should be concentrated in fostering strong collaboration between researchers, clinicians and regulatory agencies to uncover patient needs and improve translation prospects. Altogether, CRISPR constitutes a revolutionary tool for biotechnology and holds a huge potential for advancing our understanding of the IVD and its associated disorders, opening new avenues for disc regeneration.
